# A one-year follow-up study of chronic pain in community-dwelling older adults with and without neuropathic pain

**DOI:** 10.1186/s12877-017-0537-x

**Published:** 2017-07-19

**Authors:** Susanna Rapo-Pylkkö, Maija Haanpää, Helena Liira

**Affiliations:** 1 Espoo Hospital, Karvasmäentie 6, 02070 City of Espoo, Finland; 2 Unit of Primary Health Care, Helsinki University Central Hospital and Department of General Practice, University of Helsinki, Helsinki, Finland; 3 Mutual Insurance Company Etera, Helsinki, Finland; 4 Department of Neurosurgery, Helsinki University Central Hospital, Helsinki, Finland

**Keywords:** Chronic pain, Finland, Older adults, General practice, Longitudinal studies

## Abstract

**Background:**

Chronic, mostly musculoskeletal pain is common among older adults. Little is known about the prognosis of chronic pain and the neuropathic pain qualities in older adults. We studied a cohort of community-dwelling older adults, clinically assessed their pain states, classified their type of pain (nociceptive, neuropathic or combined) and followed them up for a year.

**Methods:**

At baseline, a geriatrician clinically examined all study patients and classified their type of pain in collaboration with a pain specialist. Pain, quality of life and mental health were measured by questionnaires (BPI, GDS-15, BAI and SF-36) and reassessed after 1 year.

**Results:**

Despite chronic pain, all patients from the baseline cohort continued to live independently at 1 year. A total of 92 of 106 (87%) patients returned the follow-up questionnaire. Nociceptive pain on its own was present in 48 patients, whereas 44 patients also had neuropathic pain. Most patients (96%) had several pain states at baseline, and 13 patients reported a new pain state at follow-up. On average, there were no significant changes in the pain intensity, pain interference, mood or quality of life in either group between baseline and follow-up. Changes in pain were observed at the individual level, and both intensity and interference of pain at the follow-up had a negative correlation with the baseline value.

**Conclusions:**

On average, chronic pain was persistent in our patients, but they were able to live independently despite their pain. At the individual level, both relief and exacerbation of pain were observed, supporting the notion that pain is not inevitable and unremitting among older adults.

## Background

It has been estimated that 20% of the European population suffers from chronic pain [1], affecting most often the musculoskeletal system [2]. Chronic pain impairs activities of daily living and mobility, and may predict the progression of disability in home-dwelling older persons [3]. Chronic pain can be classified according to its pathophysiology into nociceptive pain, neuropathic pain, and pain without a known somatic background [4].

Nociception is known to change with advancing age [5]. The oldest old adapt to living and coping with chronic pain [6]. In population-based studies, neuropathic pain has been more severe than other types of pain [7, 8]. Depression and anxiety appear to be more common in patients with neuropathic pain compared to those without [9]. However, it is not known whether this is true of older people. To date, there are no studies comparing cohorts of older adults with different chronic pain types in longitudinal settings, while follow-up studies on chronic pain are scarce [10, 11].

Our aim was to study and follow up a cohort of home-dwelling older adults with chronic pain. We have shown in our previous study that patients with neuropathic pain have more severe pain and greater disability than other patients with chronic pain [12]. As neuropathic pain is known to be difficult to treat and intractable in many cases, we presumed that those with neuropathic pain might have less favorable prognosis of their pain in follow-up. In this study we report the results on pain, mental health and quality of life at a 1-year follow-up by comparing patients with and without neuropathic pain.

## Methods

The municipality of Kirkkonummi (population 37,600 inhabitants in 2012) organized preventive home visits in the period 2009–2013 for older adults aged 75, 80 and 85 years who lived independently at home. Adults in the target age-group were given an opportunity for a home visit by a nurse. Among other issues, the presence of chronic (duration >3 months) pain was inquired using a one-sheet questionnaire. Those having pain with an average daily intensity of ≥4 on a numeric rating scale (NRS) during the previous week or with at least moderate interference in daily life were offered a consultation with a geriatrician (SR-P). The exclusion criteria were impaired cognitive function (MMSE <23) or impaired communication skills (aphasia or insufficient ability to speak Finnish or Swedish).

During the consultation visit, the study nurse introduced the procedure to the patient and handed four questionnaires to them. To cover the burden of pain, we chose questionnaires for intensity and interference of pain, depression, anxiety and quality of life, using the Severity and Intensity subscales of the Brief Pain Inventory (BPI) [13], the Geriatric Depression Scale (GDS-15) [14], the Beck Anxiety Inventory (BAI) [15], and the Medical Outcomes Survey Short Form (SF-36) [16], which are validated and widely used instruments in clinical studies.

### BPI

The BPI is a patient-completed numeric rating scale that assesses the severity of pain (Severity scale) and its impact on daily functioning (Interference scale). The Pain Interference Scale assesses the degree to which pain interferes with seven daily activities (general activity, mood, normal work, walking, relations with others, sleep, and enjoyment of life). The ratings are measured using 11-point numeric rating scales ranging from 0 (does not interfere) to 10 (completely interferes). The mean of these seven ratings is used to indicate the patient’s overall level of pain interference.

### GDS-15

The GDS-15 was designed as a self- or interviewer-administered screening instrument and consists of questions addressing various depressive symptoms and is of particular value for use with older patients since it has a simple yes/no format and does not rely on somatic symptoms that may be part of the normal ageing process or an associated physical illness.

### BAI

The BAI is a 21-item screening instrument designed as a general measure of anxiety and to differentiate symptoms of anxiety from symptoms of depression. The items are answered on a 4-point Likert-type scale ranging from 0 (not at all) to 3 (severe, I could barely stand it). The responses are summed to provide a score ranging from 0 to 63, with higher scores indicative of higher levels of anxiety.

### SF-36

The SF-36 is a short questionnaire with 36 items which measure eight multi-item variables: physical functioning (10 items), social functioning (two items), role limitations due to physical problems (four items), role limitations due to emotional problems (three items), mental health (five items), energy and vitality (four items), pain (two items), and general perception of health (five items). There is a further unscaled single item on changes in respondents’ health over the past year [16].

After the patient had filled in the questionnaires the geriatrician examined the patients with the aim of diagnosing the etiology of the pain state(s) and the type(s) of pain (nociceptive, neuropathic or combined). If clinically indicated, the geriatrician had an opportunity to refer the patients for laboratory or imaging investigations or for a consultation with another specialist (e.g., neurologist or orthopedic surgeon etc.). As appropriate, the geriatrician modified the pain treatment (e.g., recommendation of dose escalation or trial of another drug for alleviating pain). The GPs then followed up the modified treatment plans.

A follow-up questionnaire was sent to the patients 1 year after the visit to the geriatrician. It included questions on current pain medication and possible new pain states, and the severity and intensity subscales of the BPI, GDS-15, BAI and SF-36. Information about the diagnostic procedures and treatment of pain during the follow-up period were checked and collected from the files of each patient by the geriatrician (SR-P).

#### Statistical methods

Results are presented using means, standard deviations and frequency distributions. Statistical significance between groups was tested by the t-test, Mann-Whitney test or chi-square test. In the case of a violation of the assumptions (e.g. non-normality), a permutation-type test was used. The differences between groups in changes over the 1-year period were compared using a bootstrap-type ANCOVA with the baseline measurement as a covariate. Correlation coefficients were calculated by the Pearson method. The normality of the variables was tested using the Shapiro-Wilk W test. The statistical package used was Stata 14.0, StataCorp LP (College Station, TX, USA).

#### Ethics approval

The study protocol was approved by the Ethics Committee of the Helsinki University Central Hospital (permission 128/13/03/00/09), and written informed consent was obtained from all participants.

## Results

Altogether 92 (26 men, 28% and 66 women, 72%) of 106 patients from the original cohort replied to the follow-up postal survey, representing 87% of the baseline cohort. According to patient files, all patients from the original cohort continued to live independently at home. None of the patients in the baseline cohort had died, and the reasons for non-response to the follow-up questionnaire remain unknown.

Four patients (4%) had only one pain state and 88 (96%) patients had two or more different pain states. Nociceptive pain on its own was present in 48 (52%) patients, whereas 44 (48%) patients also had neuropathic pain. None of our patients had neuropathic pain alone. The causes of nociceptive pain were spine disorders (*n* = 55, 60%), osteoarthritis of limb joints (*n* = 38, 41%), soft tissue disorders (*n* = 28, 30%, with shoulder pain in 16), sequelae of injuries (*n* = 9, 10%), inflammatory polyarthropaties (*n* = 5, 5%), visceral pain (*n* = 5, 5%) and primary headache (*n* = 2, 2%). Neuropathic pain was due to degenerative disease of the spine causing radiculopathy (*n* = 25, 59% of the cases of neuropathic pain), peripheral nerve trauma (*n* = 7, 16%), peripheral nerve entrapment (*n* = 4, 9%), painful polyneuropathy (*n* = 4, 9%), postherpetic neuralgia (*n* = 3, 7%) and central post-stroke pain (*n* = 1, 2%).

The baseline characteristics of patients with and without neuropathic pain are presented in Table 1. The only significant difference between the groups was for gender (neuropathic pain was less common in women). Mean (SD) intensity of pain was 4.1 (2.0) in patients without neuropathic pain and 5.1 (1.8) in patients with neuropathic pain (*p* = 0.003, adjusted for age and gender). Mean (SD) interference of pain was 3.4 (2.4) in patients without neuropathic pain and 4.5 (2.3) in patients with neuropathic pain (*p* = 0.007, adjusted for age and gender). Median (IQR) follow-up time was 12 [8, 15] months in patients without neuropathic pain and 12 [8, 12] months in those with neuropathic pain.

**Table 1:** Baseline characteristics of patients without neuropathic pain and with neuropathic pain

Variable	NEP- (*N* = 48)	NEP+ (*N* = 44)	*P*-value*
Number of women, (%)	41 (85)	25 (57)	0.002
Age group, years, N (%)			0.87
75	30 (62)	29 (66)	
80	12 (25)	11 (25)	
85	6 (13)	4 (9)	
Living alone, N (%)	25 (53)	18 (41)	0.24
Duration of pain, years, (%)			0.72
< 1	8 (17)	8 (18)	
1–2	15 (31)	10 (23)	
≥ 3	25 (52)	26 (59)	
MMSE, mean (SD)	28 (2)	27 (2)	0.44
Subjective health, N (%)			0.97
Good	17 (37)	16 (36)	
Satisfactory	21 (46)	21 (48)	
Insufficient	8 (17)	7 (16)	
Subjective moving capability, n (%)			0.76
Good	16 (33)	15 (34)	
Satisfactory	18 (38)	19 (43)	
Insufficient	14 (29)	10 (23)	
Comorbidities			
Cardiovascular diseases	35 (73)	29 (66)	0.47
Musculoskeletal diseases	31 (65)	21 (48)	0.10
Endocrine diseases	22 (46)	18 (41)	0.63
Respiratory diseases	13 (27)	8 (18)	0.31
Neoplasms	6 (13)	4 (9)	0.60
Psychiatric diseases	2 (4)	0 (0)	0.49
Nervous system diseases	1 (2)	3 (7)	0.35
BAI, mean (SD)	12.6 (8.1)	13.5 (11.6)	0.68
GDS-15, mean (SD)	3.04 (2.50)	3.84 (3.02)	0.18
SF-36, mean (SD)			
Physical Component Summary	34 (12)	33 (11)	0.63
Mental Component Summary	53 (10)	52 (11)	0.57
Pain medication, N (%)	36 (75)	36 (82)	0.43
Paracetamol	29 (60)	24 (55)	0.57
NSAID (peroral)	14 (29)	19 (43)	0.16
NSAID (topical)	8 (17)	2 (5)	0.093
Mild opioid	5 (10)	7 (16)	0.54
Neuropathic pain drug^a^	3 (6)	4 (9)	0.71

NEP-, without neuropathic pain, NEP+, with neuropathic pain

*BAI* Beck Anxiety Inventory; *GDS*-*15* Geriatric Depression Scale, *SF*-*36* Medical Outcomes Survey Short Form

*Adjusted age and gender

^a^Antidepressant drug, antiepileptic drug or topical lidocaine for pain

The treatment of patients with and without neuropathic pain is presented in Table 2. Compared to baseline, medication was changed at the follow-up in 30 cases: a new drug was prescribed for 22 patients, and the dose was modified for 8 patients. Physiotherapy was recommended for 27 patients and assistive devices (orthosis, orthotic insoles, orthotic vest or collar) were recommended for 8 patients. Twelve patients were referred to a specialist consultation. There were no significant differences between the groups regarding treatments. Nine patients underwent surgery due to chronic pain (total hip replacement for 5 patients, total knee replacement for 1, lumbar decompression for 2 and nerve entrapment decompression to 1).

**Table 2:** New treatments provided to patients without neuropathic pain and with neuropathic pain during the follow-up time

Variable	NEP- (*N* = 48) N (%)	NEP+ (*N* = 44) N (%)	*P*-value
Change in medication	12 (25)	18 (41)	0.10
Assistive devices	7 (15)	1 (2)	0.061
Physiotherapy	14 (29)	13 (30)	0.97
Diagnostic procedures	7 (15)	5 (11)	0.65

At the follow-up, 13 patients reported experiencing a new type of pain. The diagnosis was osteoarthritis of limb joints in 5 patients, visceral pain in 2, painful polyneuropathy in 2, bone fracture in 2, spinal disorder in 1, and recent herpes zoster in 1.

**Fig. 1:**
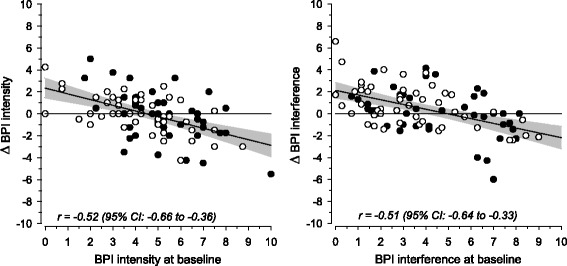
Relationship of pain at baseline and change during follow-up regarding intensity and interference. Significant correlations were between baseline and change of pain intensity and interference. ● = with neuropathic pain, o = without neuropathic pain

During the follow-up, there were no significant changes in the pain intensity in either group. The change in the intensity of pain (NRS 0–10) in those without neuropathic pain was −0.01 (95% CI: -0.49 to 0.48), *p* = 0.97 and in those with neuropathic pain −0.11 (95% CI: -0.79 to 0.58), *p* = 0.65. Differences in the changes did not differ significantly between the groups (*p* = 0.22, adjusted at baseline). The change in the interference from pain (NRS 0–10) in those without neuropathic pain was 0.76 (95% CI: 0.18 to 1.34), *p* = 0.011 and in those with neuropathic pain 0.09 (95% CI: -0.54 to 0.73), *p* = 0.77. The change in the interference from pain did not significantly differ between the groups (*p* = 0.59, adjusted at baseline). The relationship of pain at baseline and the change during follow-up regarding the intensity and interference is presented in Fig. 1. Both intensity and interference had a negative correlation with the baseline value.

Change in depression, anxiety and quality of life during the follow-up is presented in Table 3. No significant change was observed within or between the groups.

**Table 3:** Change during follow-up in depressive symptoms, anxiety and quality of life

Variable	NEP- (*N* = 48) Change (95% CI)	NEP+ (*N* = 44) Change (95% CI)	*P*-value*
GDS-15	0.5 (−0.3 to 1.3)	0.2 (−0.5 to 0.9)	0.93
BAI	−2.0 (−4.3 to 0.4)	−0.5 (−2.4 to 1.7)	0.15
SF-36			
Physical Component Summary	−2 (−4 to 1)	−2 (−4 to 1)	0.75
Mental Component Summary	−3 (−6 to 1)	−2 (−4 to 1)	0.78

NEP-, without neuropathic pain, NEP+, with neuropathic pain

*BAI* Beck Anxiety Inventory, *GDS*-*15* Geriatric Depression Scale, *SF*-*36* Medical Outcomes Survey Short Form

*Baseline adjusted

## Discussion

Chronic pain among older adults remained mostly the same at the group level in our 1-year follow-up study. This is in line with a previous longitudinal study in which three-quarters of community-dwelling older adults with pain at baseline had similar pain at 1- and 2-year follow-ups [10]. However, at the individual level we observed both relief and worsening of pain. There was a regression towards the mean in both pain intensity and interference. The variables that were extreme in the first measurement tended to be closer to the average in the second measurement. In both nociceptive and neuropathic pain groups, patients with more severe pain reported a decrease and those with milder pain reported an increase in pain. Musculoskeletal pain was similarly reported to fluctuate in the study by Karttunen and colleagues [10].

In a large community-based cohort study from the USA that included adults aged 65 or older, musculoskeletal pain was assessed annually for 6 years. One-third of adults reported pain intermittently and another third for three or more consecutive years. The authors concluded that their findings refuted the notion that pain is inevitable, unremitting or a progressive consequence of aging [11]. This is in line with our observation that some patients experience pain relief at follow-up but new pain conditions appear to the others.

None of our patients had neuropathic pain alone. Two leading causes for neuropathic pain (comprising three quarters of neuropathic pain cases in our cohort) were spinal pain and posttraumatic pain, which are typical examples of mixed pain states (combination of nociceptive and neuropathic pain). In addition, osteoatrhrosis and other degenerative musculoskeletal pain conditions causing nociceptive pain are very common in older adults, who may have also a separate neuropathic pain state.

In our cohort, patients with a neuropathic pain had higher pain intensity and interference compared with those without neuropathic pain [12]. This is in accordance with previous large population-based studies [7–9]. Reasons for this may include the refractory nature of neuropathic pain with regard to pharmacotherapy [17] and the unpredictable character of neuropathic pain; exacerbations may appear without any identifiable provoking factors, whereas nociceptive pain has clearer provoking factors (e.g., it is possible to control the pain of knee osteoarthritis by restricting activity) [18].

In spite of chronic pain, our patients did not have major mental health problems, and their anxiety and depression scores remained low. This may reflect their ability to cope with pain and to adapt to restrictions caused by conditions causing pain [6, 18]. Most of the patients in both groups had experienced pain over 3 years which also may have significant impact of coping with pain.

Our study was not designed to assess the effects of different pain management options. The GPs provided management to the patients according to their own judgment. The decisions were based on clinical practice, guidelines and individual consideration of the usefulness of treatments. Management of our patients included modification of pharmacotherapy, physiotherapy, assistive devices, and referral to surgery or specialist consultation.

The strength of our study is that our cohort consisted of a population-driven sample of home-dwelling older adults that were clinically studied in detail at baseline. A pain specialist (MH) was consulted to make sure the pain states were correctly classified. The follow-up was performed with mailed questionnaires after approximately 1 year.

The limitations of the study include the selected cohort group instead of a population-based group of subjects and the limited size of the study group, which diminish the possibility to generalize our results. Other limitations consist of the relatively short follow-up time of chronic pain and the follow-up using only questionnaires; control visits might have provided more detailed information. We recommend that researchers aim at population-based studies with long-term follow-up and face-to-face control visits in the future.

## Conclusions

We conclude that on average, chronic pain was persistent but fluctuating in our patients at the 1-year follow-up and no significant changes were noticed between neuropathic and non-neuropathic pain groups. At the individual level, both relief and exacerbation of pain was observed, supporting the notion that pain is not inevitable or unremitting among older adults. The mental wellbeing of the patients remained good, and they were able to continue living independently despite their pain.

## Abbreviations

BAI: Beck Anxiety Inventory
BPI: Brief Pain Inventory
GDS: Geriatric Depression Scale
GP: General practitioner
MMSE: Mini-mental state examination
NRS: Numeric rating scale
NSAID: Nonsteroidal anti-inflammatory drugs
SF-36: Medical Outcomes Survey Short Form
